# Supplementary material for: Biomimetic generation of the strongest known biomaterial found in limpet tooth
Source: Nat Commun. 2022 Jul 7;13:3753. doi: 10.1038/s41467-022-31139-0 (PMC9263180; doi:10.1038/s41467-022-31139-0)
Supplement: Supplementary file 1 — Supplementary Information [file 41467_2022_31139_MOESM1_ESM.pdf]

Biomimetic generation of the strongest known biomaterial found in limpet tooth

SUPPLEMENTARY DATA.

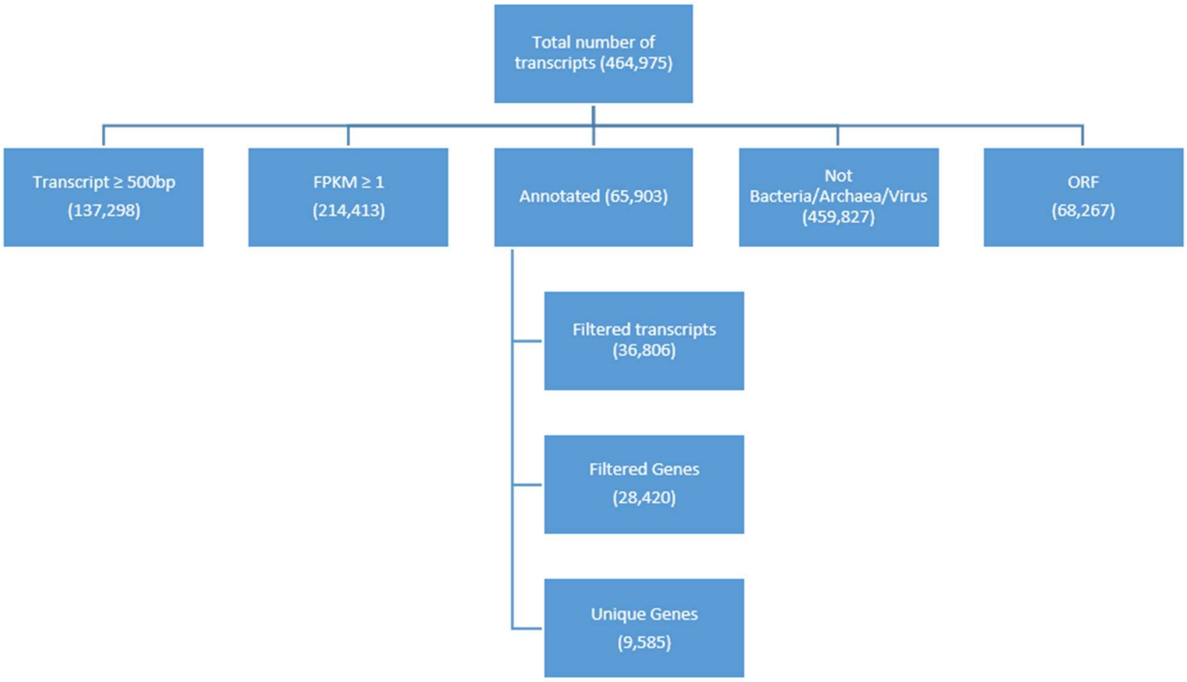

Supplementary Figure 1a: **Workflow of transcript filtering for the identification of genes within the transcriptome assembly.**

Transcripts were filtered to remove those most closely matching a gene from Bacteria, Virus or Archaea, transcripts with no identified open reading frame, transcripts with a maximum abundance less than 1 across all samples, and transcripts less than 500bp in length. All genes remaining following filtering were used for differential expression analysis regardless of whether they were annotated.

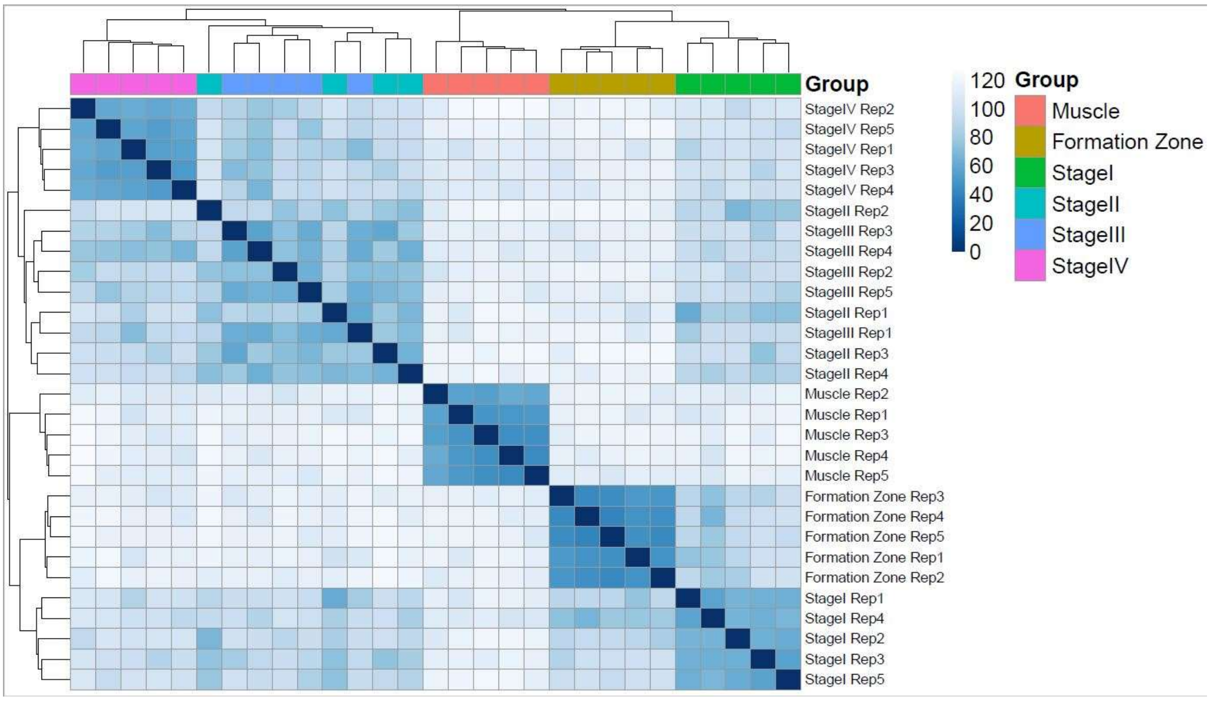

Supplementary Figure 1b: **Sample correlation matrix for the limpet RNAseq experiment.**

Samples were clustered based on their pairwise Euclidean distance in a hierarchical clustering procedure, such that samples with gene expression profiles more similar to one another produce a distance score closer to 0 (blue). Clusters of similar transcript expression signatures can clearly be seen, specifically for samples within replicate groups, and for Stage II-IV of the radula.

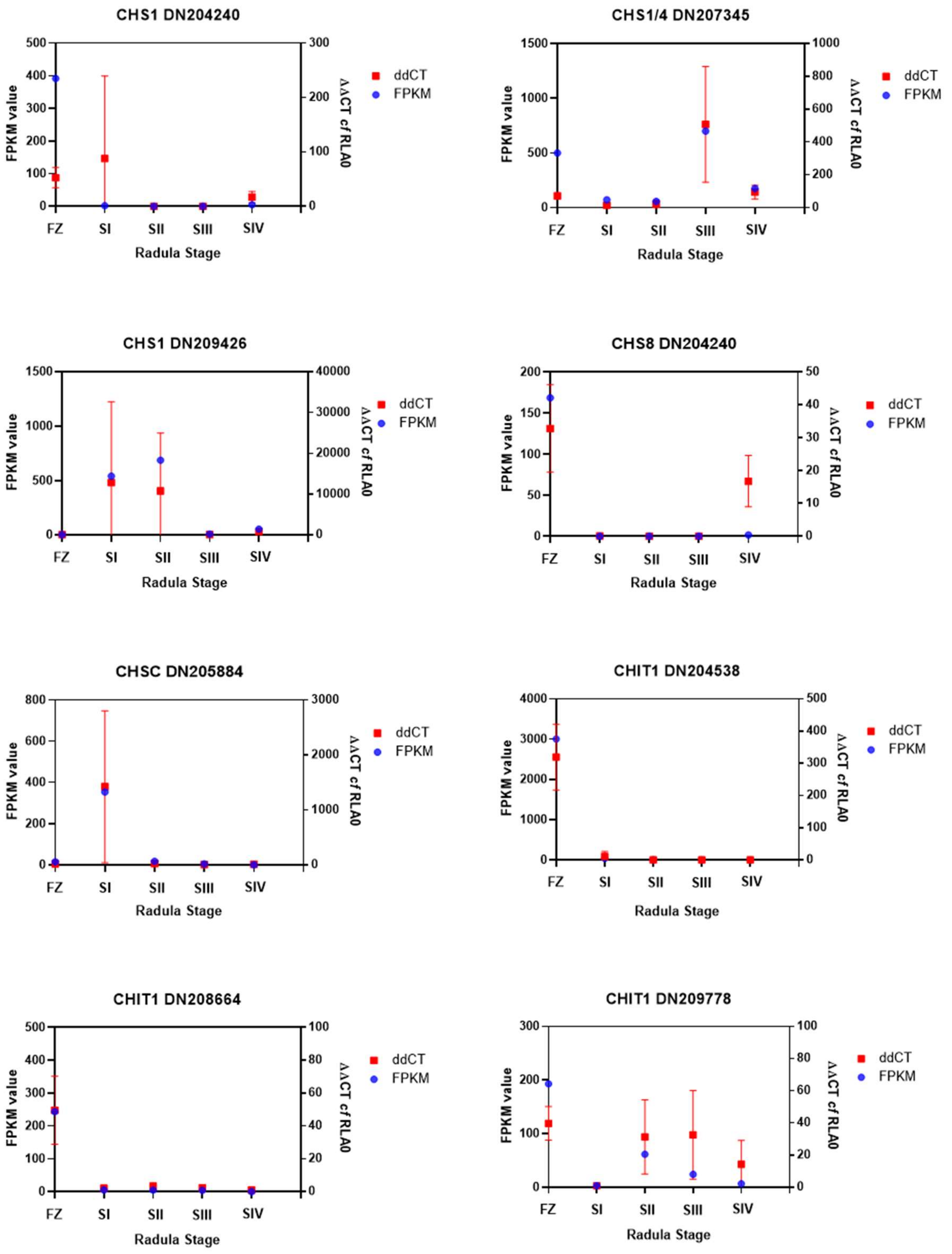

Supplementary Figure 1c: qPCR confirmation of FPKM values for chitin synthase and chitinase associated genes.

FPKM values (blue) and ddCT qPCR values (red) for chitin synthase and chitinase associated genes are shown. Points (and associated error bars for ddCT) represent the mean and standard deviation respectively over 3 replicates.

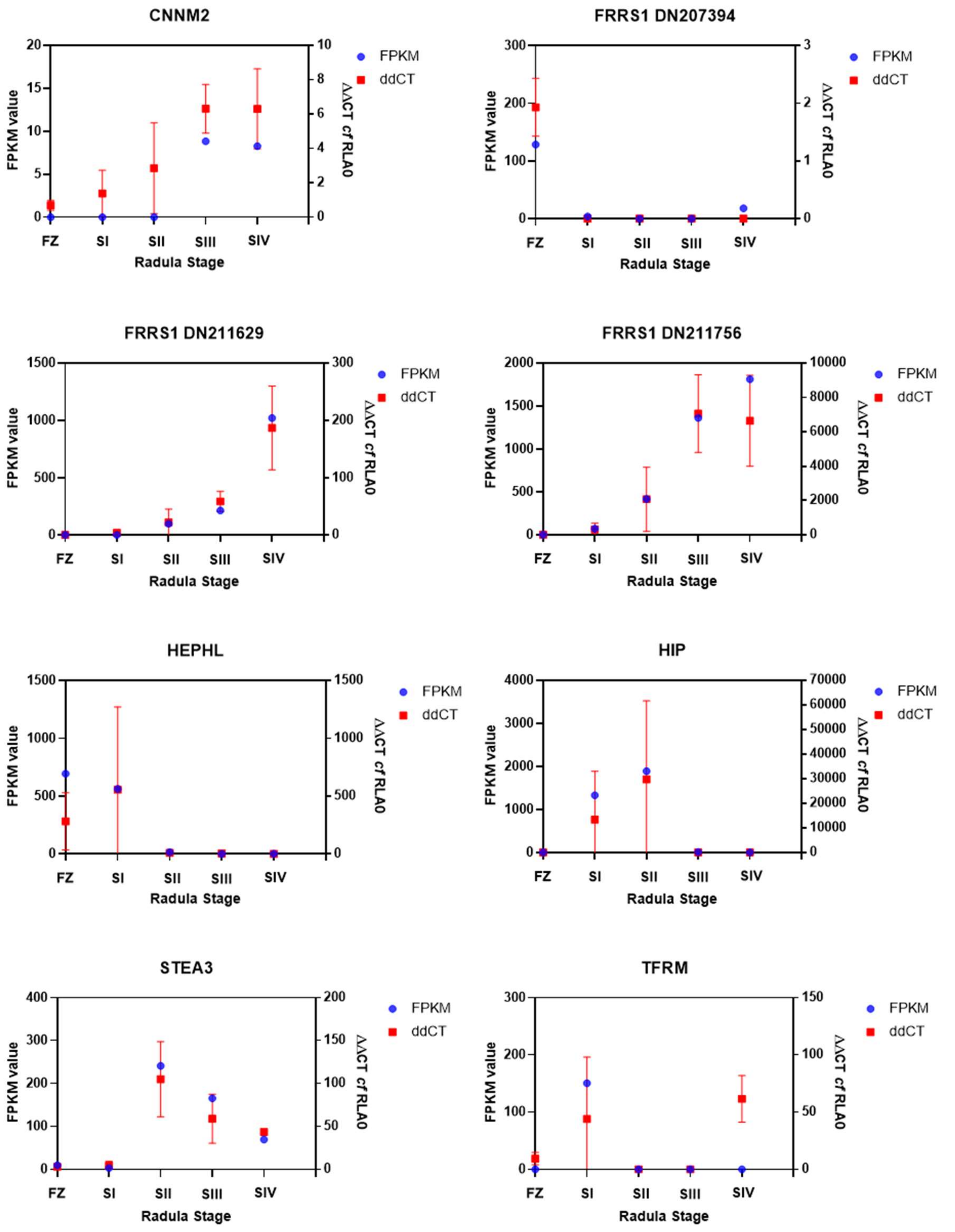

Supplementary Figure 1d: qPCR confirmation of FPKM values for iron associated genes.

FPKM values (blue) and ddCT qPCR values (red) for iron associated genes are shown. Points (and associated error bars for ddCT) represent the mean and standard deviation respectively over 3 replicates.

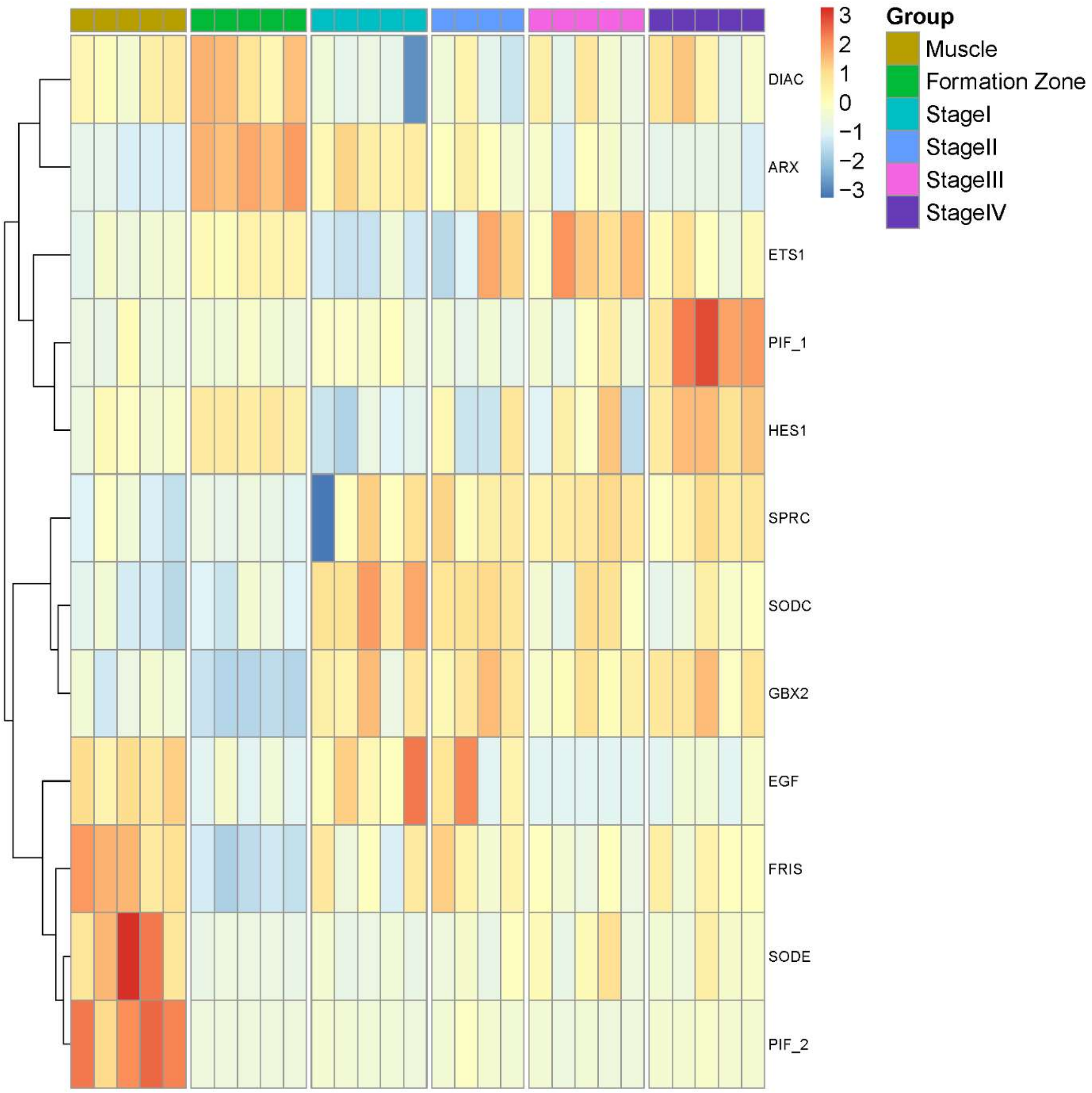

Supplementary Figure 1e: **Genes associated with radula formation**.

Heat map showing the mean abundance (z-score normalised log FPKM for RNAseq) for genes associated with radula formation across the limpet muscle, Formation Zone and radula transcriptomes.

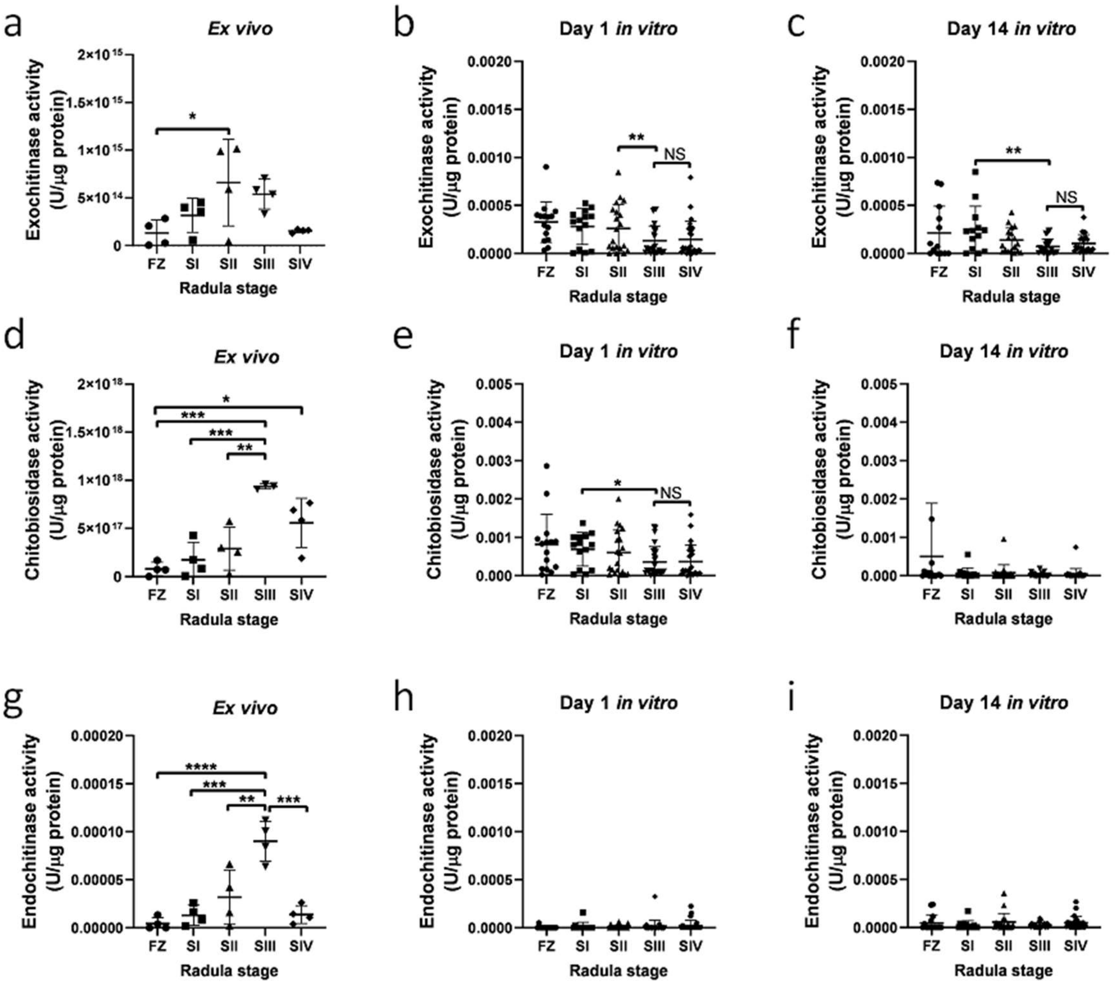

Supplementary Figure 2: **Exochitinase activity *ex vivo* and *in vitro*.**
a. Exochitinase activity in radula stages (FZ to SIV) *ex vivo* (* p = 0.0434), one-way ANOVA with Tukey's post-hoc test); **b.** cell populations isolated from specific radula stages after 1 day *in vitro* (SII v SIII, ** p = 0.002, univariate analysis with Tukey's post-hoc test); **c.** cell populations isolated from radula stages after 14 days *in vitro* (SI v SIII, ** p = 0.001801, univariate analysis with Tukey's post-hoc tests). Chitobiosidase activity in: **d.** radula stages *ex vivo* (FZ v SIII, *** p = 0.0002; SI v SIII, *** p = 0.0006; SII v SIII, ** p = 0.0029; FZ v SIV, * p = 0.0161, one-way ANOVA with Tukey's post-hoc test); **e.** cell populations isolated from radula sections after 1 day *in vitro* (SI v SIII * p = 0.030946, Univariate analysis with Tukey's post-hoc test); **f**. cell populations isolated from radula sections after 14 days *in vitro*. Endochitinase activity in: **g.** radula stages *ex vivo* (FZ v SIII **** p < 0.0001; SI v SIII *** p = 0.0001; SII v SIII ** p = 0.0018; SIII v SIV *** p = 0.0001; one-way ANOVA with Tukey's post-hoc test).; **h.** cell populations isolated from radula stages after 1 day *in vitro*; **i**. cell populations isolated from radula stages after 14 days *in vitro*. For *ex vivo* experiments **a, d,** and **g** n = 4 independent biological replicates with one measurement per limpet. For *in vitro* experiments FZ n = 15, SI n = 14, SII n = 20, SIII n = 26, SIV n = 26 across three independent donor limpet radulae. Data are presented with individual data points and mean with standard deviation error bars.

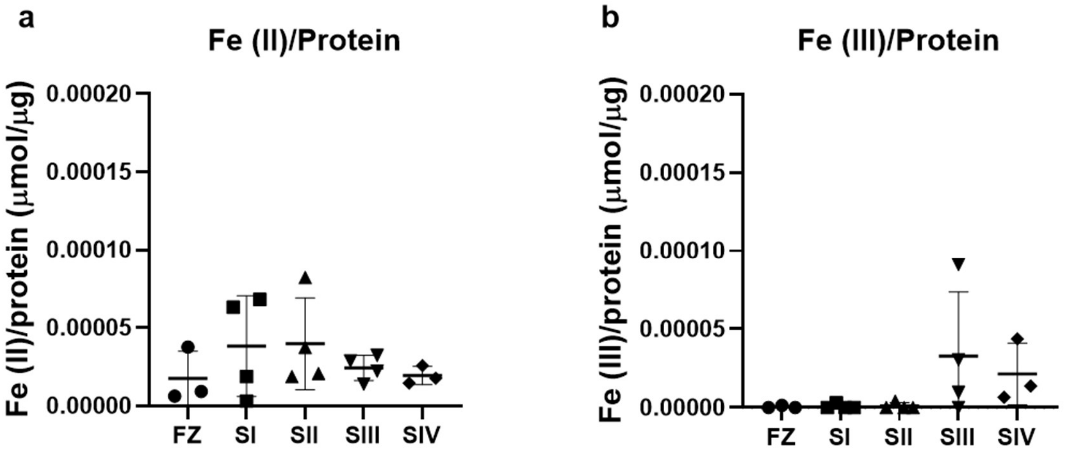

Supplementary Figure 3: **Iron in radula sections *ex vivo*.**

a. Fe(II) and **b.** Fe(III) normalised to protein in radula sections *ex vivo.* For **a** and **b,** n = 4 independent biological replicates with one measurement per limpet. Data are presented with individual data points and mean with standard deviation error bars.

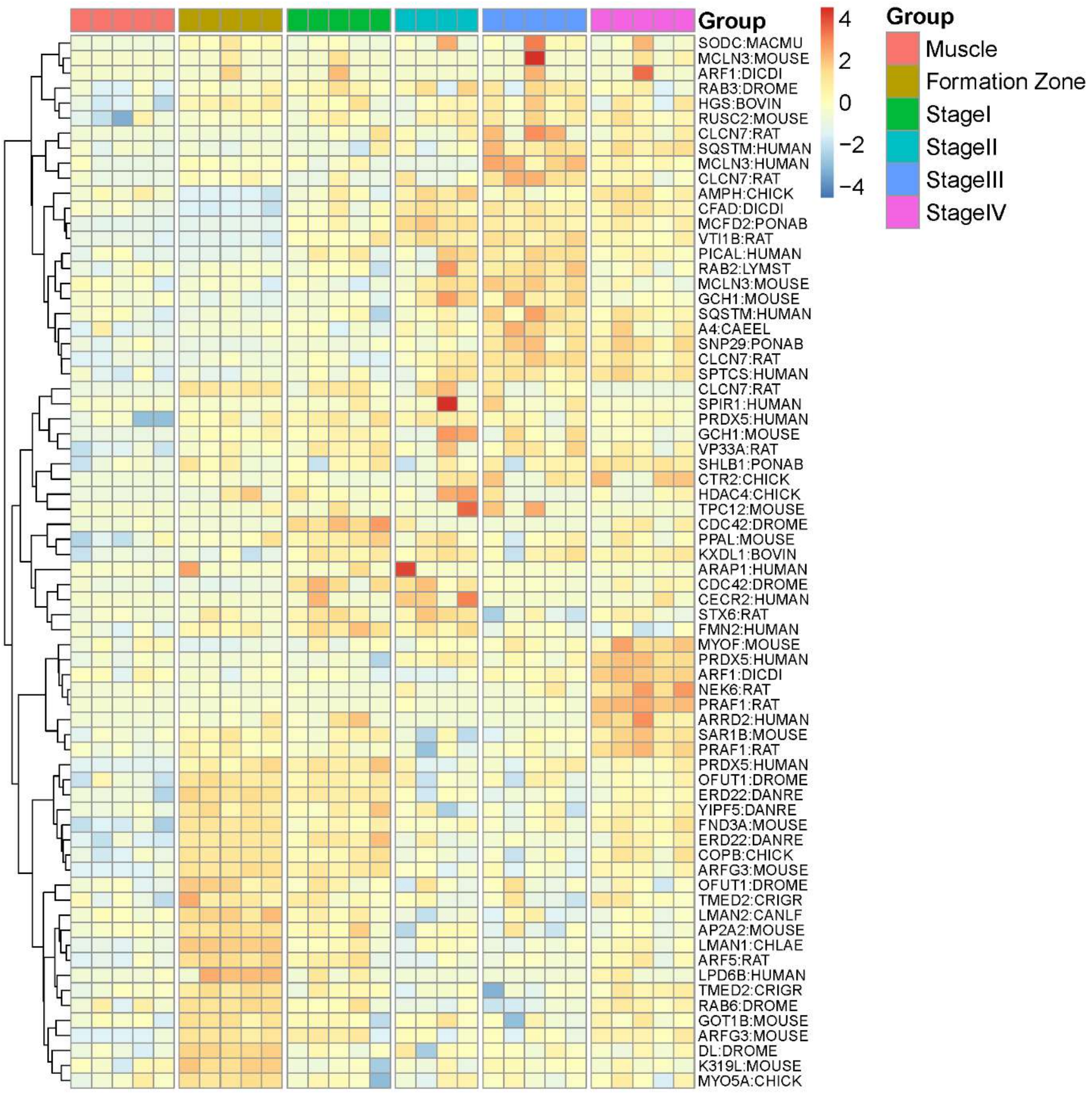

Supplementary Figure 4: Orthologous genes associated with vesicle transport.

Heat map showing the abundance (z-score normalised log FPKM for RNA seq) across the limpet muscle, Formation Zone and radula stages' transcriptomes for orthologous genes associated with vesicle transport.

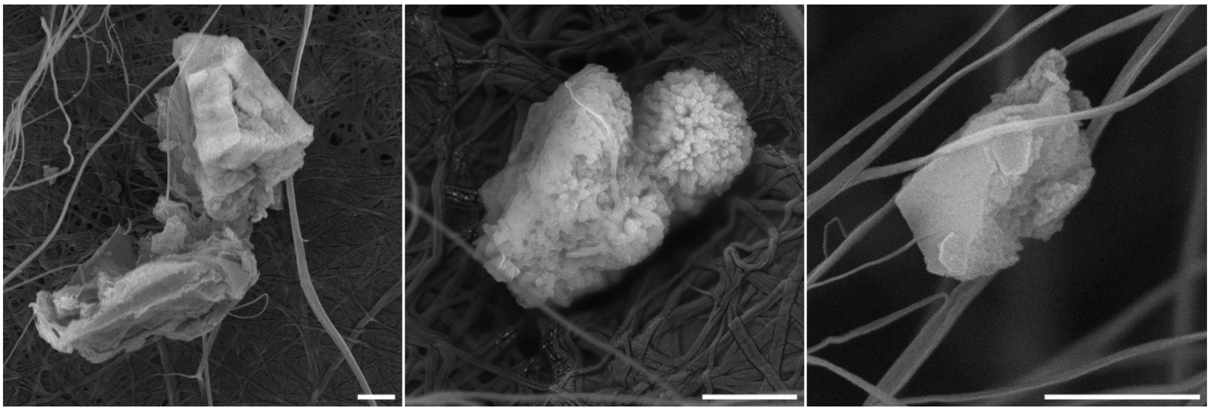

Supplementary Figure 5: **Chitin with biogenic iron crystals.**

SEM images of biogenic iron crystal structures generated within chitin fibres treated with cell conditioned media from cultures of stage III/IV limpet cells (*n* = 1, scale bar = 1 μm).

Section	Limpet replicate	Total Raw PE Reads	%GC	%Q20	RIN	Total Trimmed PE Reads	Total Mapped Reads	Percent Mapped Reads
Muscle	Limpet 1	30570214	55.14%	95.58%	6.5	30453376	23032976	75.34%
Muscle	Limpet 2	25691797	52.49%	95.18%	6.4	25574211	19408074	75.54%
Muscle	Limpet 3	25708336	51.38%	94.79%	6.4	25589308	19603827	76.25%
Muscle	Limpet 4	27296348	52.19%	94.78%	6.7	27191085	21243138	77.82%
Muscle	Limpet 5	30275662	55.51%	95.77%	6.7	30173302	22909569	75.67%
Formation Zone	Limpet 1	31375355	40.69%	96.06%	6.5	31238711	16355607	52.13%
Formation Zone	Limpet 2	32654837	40.97%	96.98%	6.7	32510373	16982667	52.01%
Formation Zone	Limpet 3	28435575	40.17%	95.24%	6.5	28216702	16411993	57.72%
Formation Zone	Limpet 4	30540573	40.70%	95.88%	5.3	30399274	16616105	54.41%
Formation Zone	Limpet 5	28061885	40.34%	96.55%	6.9	27959724	16400542	58.44%
Stage I	Limpet 1	28336255	43.80%	95.42%	6.1	28152554	19872049	70.13%
Stage I	Limpet 2	29488127	40.43%	96.46%	6.6	29220457	19562159	66.34%
Stage I	Limpet 3	26379183	40.50%	95.49%	6.3	26218337	17939042	68.00%
Stage I	Limpet 4	27229706	41.44%	95.61%	6.3	27076655	19176132	70.42%
Stage I	Limpet 5	31160241	43.40%	96.01%	3.8	30917529	20615719	66.16%
Stage II	Limpet 1	29124893	44.81%	95.90%	4.7	28970154	19633793	67.41%
Stage II	Limpet 2	27845971	48.02%	94.77%	8	27610792	18761160	67.37%
Stage II	Limpet 3	25907078	47.10%	94.29%	6.2	25699657	18074995	69.77%
Stage II	Limpet 4	33307627	51.04%	95.57%	5.1	32850814	23892971	71.73%
Stage II	Limpet 5	30192016	48.92%	95.40%	7.6	29722571	17868319	59.18%
Stage III	Limpet 1	31972008	50.75%	95.61%	6.3	31797232	23020407	72.00%

Stage III	Limpet 2	27321940	50.35%	94.49%	7.1	27119545	19646518	71.91%
Stage III	Limpet 3	31653218	46.37%	95.60%	6.9	31508137	22182851	70.08%
Stage III	Limpet 4	31784782	46.83%	96.44%	7.3	31634192	22756335	71.60%
Stage III	Limpet 5	31402707	49.37%	97.04%	7.1	31305078	22829602	72.70%
Stage IV	Limpet 1	25524530	42.89%	95.00%	4.8	25358744	16875419	66.11%
Stage IV	Limpet 2	28081279	44.02%	95.20%	6.9	27938750	17509071	62.35%
Stage IV	Limpet 3	26591472	44.20%	95.11%	5.8	26454881	17983408	67.63%
Stage IV	Limpet 4	27234251	47.69%	95.26%	5.7	27110413	18554453	68.13%
Stage IV	Limpet 5	30349635	44.21%	96.25%	6.9	30253584	18390780	60.60%

Supplementary Table 1: Sample statistics for NGS samples used in this study.

For each sample, the total number of raw paired reads, the number of trimmed paired reads, and the number and percentage of paired reads that map back to the assembled transcriptome are shown. In addition, the GC percentage (%GC), percentage of reads with average quality score greater than 20 (%Q20), and the RNA Integrity Number (RIN) are shown.

TRINITY accession number	Gene name	Corresponding protein	F Primer	R primer
DN204538_c2_g1_i3	CHIT1	Chitotriosidase-1	CGTGGCAAATACCCACTCCT	GGACGCCGACTTCTTTTTGG
DN208664_c1_g2_i1	CHIT1	Chitotriosidase-1	GTGGCATCGTACCAAACCCT	TGTTCGTGTTCGTGGGGTAG
DN209778_c0_g2_i9	CHIT1	Chitotriosidase-1	TTGCGGCGAATACGAAACTC	GTCCACCCAGTCCCCTTACT
DN209426_c1_g1_i1	CHS1	Chitin synthase 1	TACGCCAAACGCGGTTCTAA	TGGGCACGACAACTCCTAAC
DN207345_c1_g1_i2	CHS1/4	Chitin synthase 1	GCTTCCCATCTACGCCTTGT	CTGCTCCTCCTCCGACATTC
DN204240_c1_g3_i1	CHS1	Chitin synthase 1	GTCGGCAAATCAGAAACGCA	CCTGCGGATGAAAACATGCC
DN204240_c1_g1_i5	CHS8	Chitin synthase 8	TGGTCCTTTCGATGCCGATT	AAAGAGAGCCACCAAGCCAA
DN205884_c0_g1_i6	CHSC	Chitin synthase C	CATGGTCCGCTTCAGCTCTA	AAACCAGATGACGACGACCC
DN205993_c2_g1_i3	CNNM2	Metal transporter CNNM2	TTGAGGCTGGACCGTTTACC	TAGTAGTTCGTCGTGCTGCC
DN211756_c2_g1_i3	FRRS1	Putative ferric-chelate reductase 1	ATCCCCAACGAACACCAAGT	CCGTTTCGGTGCGTTATCAG
DN207394_c6_g2_i2	FRRS1	Putative ferric-chelate reductase 1	GGCTGCTGAAGTCAAACAGG	CATGCAGATTCGTGCCGTAG
DN211629_c0_g1_i2	FRRS1	Putative ferric-chelate reductase 1	TCATCTGAGCGAAGAGCGTT	CGCTTTGTGTCCAGTAGGGT
DN201234_c1_g1_i1	HEPHL	Hephaestin-like protein	CGCAGCTATGGAGGTTGAGT	TCCACGAATGACAGGGGTTG
DN213713_c2_g2_i1	HEPHL	Hephaestin-like protein	AGCACGAGGTTGAGAGAACC	CCAGGTGGAACTCGGTCATC

DN213138_c7_g1_i2	HIP	Heavy metal-binding protein HIP	CCGAGTCATATCGCTGGAGG	CCAGTGAGTGTTCTGAGCGA
DN193077_c4_g1_i1	RLA0	60S acidic ribosomal protein P0	CTGCCAAAGCCAATGCTCTC	CTCCGACACGATCTCCCTCT
DN166555_c1_g1_i1	RS13	40S ribosomal protein S13	AGGGGTTGACACCATCACAA	GCATTCTTGTCCTTTCGGCA
DN200471_c2_g1_i4	STEA3	Metalloreductase STEAP3	ATACAGGGCGCTAACATCCG	CGTCCCTGTGCTCCTAACAA
DN194805_c2_g1_i2	TRFM	Melanotransferrin	AAGGACGCCAGCAACTACAA	AGGTATTCAGCACCAAGCCA
DN201234_c1_g1_i1	HEPHL	Hephaestin-like protein	CGCAGCTATGGAGGTTGAGT	TCCACGAATGACAGGGGTTG
DN213713_c2_g2_i1	HEPHL	Hephaestin-like protein	AGCACGAGGTTGAGAGAACC	CCAGGTGGAACTCGGTCATC

Supplementary Table 2: Trinity accession numbers, gene names, corresponding proteins, and primer sequences used to confirm differential gene expression data.
