# Supplementary material for: Biomimetic generation of the strongest known biomaterial found in limpet tooth
Source: Nat Commun. 2022 Jul 7;13:3753. doi: 10.1038/s41467-022-31139-0 (PMC9263180; doi:10.1038/s41467-022-31139-0)
Supplement: Supplementary file 3 — Description of Additional Supplementary Files [file 41467_2022_31139_MOESM3_ESM.pdf]

Description of Additional Supplementary Files

File Name: Supplementary Movie 1
Description: A recording showing FZ and radula isolation from limpet.

File Name: Supplementary Movie 2
Description: Animation showing XCT of whole mount limpet with radula *in situ*; radula colouration highlights change in material density.

File Name: Supplementary Movie 3
Description: Animation showing XCT of mineralisation within lab grown limpet teeth.

File Name: Supplementary Data 1
Description: Table showing the annotation results for all transcripts in the transcriptome assembly.

File Name: Supplementary Data 2
Description: Normalised abundance of all transcripts for each sample, measured as fragments per kilobase mapped (FPKM).

File Name: Supplementary Data 3
Description: Gene expression results from DESeq2 for Formation Zone vs. muscle, stage I vs. muscle, stage II vs. muscle, stage III vs. muscle and stage IV vs. muscle.
