# Supplementary material for: Biomimetic generation of the strongest known biomaterial found in limpet tooth
Source: Nat Commun. 2022 Jul 7;13:3753. doi: 10.1038/s41467-022-31139-0 (PMC9263180; doi:10.1038/s41467-022-31139-0)
Supplement: Supplementary file 10 — Reporting Summary [file 41467_2022_31139_MOESM10_ESM.pdf]

Corresponding author(s): Dariusz C Gorecki

Last updated by author(s): May 11, 2022

Reporting Summary

Nature Portfolio wishes to improve the reproducibility of the work that we publish. This form provides structure for consistency and transparency in reporting. For further information on Nature Portfolio policies, see our Editorial Policies and the Editorial Policy Checklist.

Statistics

For all statistical analyses, confirm that the following items are present in the figure legend, table legend, main text, or Methods section.

n/a	Confirmed	
☐	☒	The exact sample size (*n*) for each experimental group/condition, given as a discrete number and unit of measurement
☐	☒	A statement on whether measurements were taken from distinct samples or whether the same sample was measured repeatedly
☐	☒	The statistical test(s) used AND whether they are one- or two-sided<br>*Only common tests should be described solely by name; describe more complex techniques in the Methods section.*
☐	☒	A description of all covariates tested
☐	☒	A description of any assumptions or corrections, such as tests of normality and adjustment for multiple comparisons
☐	☒	A full description of the statistical parameters including central tendency (e.g. means) or other basic estimates (e.g. regression coefficient) AND variation (e.g. standard deviation) or associated estimates of uncertainty (e.g. confidence intervals)
☐	☒	For null hypothesis testing, the test statistic (e.g. *F*, *t*, *r*) with confidence intervals, effect sizes, degrees of freedom and *P* value noted<br>*Give P values as exact values whenever suitable.*
☒	☐	For Bayesian analysis, information on the choice of priors and Markov chain Monte Carlo settings
☐	☒	For hierarchical and complex designs, identification of the appropriate level for tests and full reporting of outcomes
☒	☐	Estimates of effect sizes (e.g. Cohen's *d*, Pearson's *r*), indicating how they were calculated

Our web collection on statistics for biologists contains articles on many of the points above.

Software and code

Policy information about availability of computer code

Data collection	RNA sequencing: RNA sequencing data were generated using the Illumina HiSeq 2500 sequencer (Reagent Kit v1.5) to produce paired-end 100 bp sequences. Base calling was performed using the HiSeq Control and Real-Time Analysis Software (RTA3 v2.2.58). X-ray micro computed tomography (XCT): Samples were imaged using high-resolution XCT (ZEISS Xradia Versa 520 Versa, Carl Zeiss X-ray Microscopy, Pleasanton, CA, USA) set to operate at 50 kV and 4 W for all samples. Isotropic voxel sizes ranged from 1.5 to 9.83 µm. Projection images were acquired over 360° at equal intervals. Scanning Electron Microscopy (SEM): Samples were dried at critical point with liquid CO2 using a Leica EM CPD300, then mounted on carbon adhesive tabs and sputter coated using a gold-palladium target in a Quorum 150 Coating Unit. Specimens were imaged by SEM using a Tescan Mira 3 with a Schottky field emission gun operated in high vacuum mode at an acceleration voltage of 15kV. Raman spectroscopy: Samples were analysed using Renishaw inVia Qontor Raman microscope, air cooled 300 mW 785 nm laser at 1% power under 50x magnification for 5 accumulations at 10 seconds exposure. Areas of iron accumulation were identified using Prussian blue staining. Raman data was collected from areas adjacent to the most intense staining. Atomic force microscopy (AFM): Samples were attached to nickel stubs for AFM analysis using a Multi-Mode/NanoScope IV scanning probe microscope (Bruker, Santa Barbara, CA, USA). A single silicon probe (t = 3.6–5.6 µm, l = 140–180 µm, w = 48–52 µm, v0 = 312.73 kHz, k = 12–103 N m-1, R < 7 nm; model: OTESPA, Bruker, France) was used for all the AFM measurements. Force vs. distance curves were obtained (1000 nm away from the surface to a 100 nm cantilever deflection trigger against the samples. Approach and retraction force curves (100 each) were obtained in a 10 x 10 area, each point separated by 1000 nm (approach and retraction speed = 3.69 um s-1) was used. The sensor response (47.02 nm V-1) was obtained on the glass surface and no subsequent AFM laser nor photodetector repositioning was made.
Data analysis	RNA sequencing: Quality control of the raw reads was performed using FastQC v0.11.7. Read trimming was performed using Trim Galore v0.4.4 using the following parameters "--illumina -q 20 --stringency 5 -e 0.1 --length 20 --trim-n" to remove Illumina adapter sequence contamination, and to trim reads for ambiguous or low-quality base calls. Reads were combined across the data set and used to generate a putative transcriptome assembly using Trinity v2.5.1 with parameters "--seqType fq --max_memory 100G --CPU 24 --min_contig_length 200 --min_kmer_cov 1 -- SS_lib_type RF --verbose --full_cleanup". TransDecoder v5.0.2 (using default parameters) was used to identify open reading frames (ORF) of 100 amino acids or more within transcripts, and putative protein amino acid sequences were produced. Transcripts were annotated against the Universal Protein Knowledge Base (UniProtKB) SwissProt database (2018_01) using BLAST v2.7.1 with an E value

cutoff of 1e-5, either at the protein level by taking the TransDecoder-derived peptide sequence (using "blastp") or from the translated nucleotide sequence directly if no ORF was identified (blastx). Additional annotation was performed against the Protein family (Pfam) database (v31.0) using HMMER (v3.1b2), providing data from the Clusters of Orthologous Groups of proteins (eggNOG) database, the Kyoto Encyclopaedia of Genes and Genomes (KEGG) database, and Gene Ontology (GO) database. Results were collated into a single output table using Trinotate v3.02 (http://trinotate.github.io/).
Differential expression analyis: Transcript abundance was estimated for individual libraries against the assembled transcriptome using Kallisto v0.43.1 with parameters "--rf-stranded". Transcripts were filtered to keep only those greater than 500 bp in length with an identified open reading frame (ORF) and a FPKM abundance score above 1 in at least one sample. In addition, transcripts most closely matching Archaeal, Bacterial or Viral species were removed. Differential expression analysis between the different tissues was conducted using the DESeq2 package v1.20.0 in R v3.5.1. Resulting p-values were adjusted for multiple testing using the Benjamini and Hochberg false discovery rate correction.
XCT: Visualisation of the reconstructed volumes was carried out using TXM3DViewer (Carl Zeiss X-ray Microscopy, Pleasanton, CA, USA and VGStudio 2.2 , Volume Graphics, Germany).
SEM: Images were captured at a working distance of 15mm in secondary electron mode. Elemental composition of particles was examined by energy dispersive X-ray spectroscopy (EDS) using an Oxford Instruments XMax 50 EDS detector and INCA software (version 5.05).
Raman spectroscopy: Spectral libraries used were the L60000 complete ST Japan library (Sept 2020, 16898 spectra) and the Renishaw Minerals and Inorganic materials library (Sept 2020, 1000 spectra).
AFM: Typical approach force curves (n = 5) were converted to text files using NanoScope Analysis software (V 1.4, Bruker). Force curves were aligned with those from the glass (hard reference) substrate to achieve a common tip-sample contact point. Reduced moduli were obtained from the linear portion of force vs. indentation3/2 plots according to Hertzian mechanics, modelling the AFM tip as a spherical indenter. Young's moduli were calculated from the reduced moduli assuming a Poisson ratio of 0.5.
Statistics: Statistical tests were carried out in GraphPad Prism 8 and IBM SPSS Statistics 25 software packages. Shapiro Wilk and Kolmogorov-Smirnov normality tests and ROUT outliers tests were carried out in GraphPad Prism. Where more than two groups were compared, significance was determined using a Univariate Analysis of Variance and differences between individual treatment groups were determined using either Tukey or Dunnett's post-hoc tests.

For manuscripts utilizing custom algorithms or software that are central to the research but not yet described in published literature, software must be made available to editors and reviewers. We strongly encourage code deposition in a community repository (e.g. GitHub). See the Nature Portfolio guidelines for submitting code & software for further information.

Data

Policy information about availability of data

All manuscripts must include a data availability statement. This statement should provide the following information, where applicable:
- Accession codes, unique identifiers, or web links for publicly available datasets
- A description of any restrictions on data availability
- For clinical datasets or third party data, please ensure that the statement adheres to our policy

All raw sequence read level data analyze during this study are available from the NCBI Sequence Read Archive (SRA) under Bioproject PRJNA566106 (https://www.ncbi.nlm.nih.gov/bioproject/PRJNA566106). The transcriptome assembly is available from the NCBI Transcriptome Shotgun Assembly Archive (TSA) under accession GHWI00000000 (https://www.ncbi.nlm.nih.gov/nuccore/GHWI00000000.1). The full annotation for the assembled transcriptome has been deposited in FigShare under a CC0 license under DOI https://doi.org/10.6084/m9.figshare.15035988. Transcripts were annotated against the Universal Protein Knowledge Base (UniProtKB) curated SwissProt database release 2018_01 (https://www.uniprot.org/uniprot/?query=reviewed:yes) using BLAST v2.7.1, and the Protein family (Pfam) database version 31.0 (https://pfam.xfam.org/) using HMMER (v3.1b2). This provided additional data annotation from the Clusters of Orthologous Groups of proteins (eggNOG) database, the Kyoto Encyclopaedia of Genes and Genomes (KEGG) database, and Gene Ontology (GO) database. For Raman spectroscopy, spectral libraries used were the L60000 complete ST Japan library (Sept 2020, 16898 spectra) and the Renishaw Minerals and Inorganic materials library (Sept 2020, 1000 spectra). All other data generated or analysed during this study are included in this published article (and its Supplementary information files).

Field-specific reporting

Please select the one below that is the best fit for your research. If you are not sure, read the appropriate sections before making your selection.

☒ Life sciences ☐ Behavioural & social sciences ☐ Ecological, evolutionary & environmental sciences

For a reference copy of the document with all sections, see nature.com/documents/nr-reporting-summary-flat.pdf

Life sciences study design

All studies must disclose on these points even when the disclosure is negative.

Sample size	The choice of 5 replicates per condition was based on a power calculation designed to give approximately 90% power to detect changes of 2-fold or greater with an alpha threshold of 0.05. This power calculation is based on average gene expression values and estimates of variance from RNA seq of oyster trocophores taken from http://www.oysterdb.com (accessed May 2017), which showed gene expression results over 5 replicates, with mean = 171.44 and standard deviation = 78.32.
Data exclusions	Of the ten limpets selected for the purposes of RNA sequencing, only the five with the highest quality RNA extracted across the different tissues were used. Otherwise, no data were excluded
Replication	All analyses were performed in at least 3 biological replicates (individual limpets) or in cells from at least three limpets. For the purposes of RNA sequencing, radulae were isolated from ten female limpets with a shell diameter across the widest point of 40-48 mm, and the five with

	the highest quality RNA extracted across the different tissues were used. All data reported in the manuscript have been reproduced.
Randomization	To prevent biases and remove sources of extraneous variation similar sized individuals were identified, then selected at random from those available. Groups compared in differential gene expression analyses contained extracted tissue from all five limpets in the study, so no randomization of groups was required. For chitin scaffold mineralization, chitin disks were cut from the same sheet and randomly allocated to mineralization or control groups.
Blinding	In AFM comparisons the experimenter was blinded regarding the identity of the samples analyzed. Blinding was unnecessary for the RNA sequencing data analysis, as groups were formed from extracted tissues from all limpets used in the study. No treatment groups were used.

Reporting for specific materials, systems and methods

We require information from authors about some types of materials, experimental systems and methods used in many studies. Here, indicate whether each material, system or method listed is relevant to your study. If you are not sure if a list item applies to your research, read the appropriate section before selecting a response.

Materials & experimental systems

n/a	Involved in the study
☒	☐ Antibodies
☒	☐ Eukaryotic cell lines
☒	☐ Palaeontology and archaeology
☐	☒ Animals and other organisms
☒	☐ Human research participants
☒	☐ Clinical data
☒	☐ Dual use research of concern

Methods

n/a	Involved in the study
☒	☐ ChIP-seq
☒	☐ Flow cytometry
☒	☐ MRI-based neuroimaging

Animals and other organisms

Policy information about studies involving animals; ARRIVE guidelines recommended for reporting animal research

Laboratory animals	Study did not involve laboratory animals.
Wild animals	Teeth of the common limpet (Patella vulgata) are the object of this study and so limpets were used in this project. Common limpet is not a protected species. Limpets were collected from the rock bed in the intertidal zone (Portsmouth, UK), placed on ice and processed on return to the laboratory. All chilled anesthetized limpets were dissected within approx. 30 minutes post collection.
Field-collected samples	Study did not involve field-collected samples.
Ethics oversight	University of Portsmouth Animal Welfare and Ethical Review Body (AWERB) approval (821B)

Note that full information on the approval of the study protocol must also be provided in the manuscript.
